# Efficacy and Safety of Novel Twincretin Tirzepatide, a Dual GIP/GLP-1 Receptor Agonist, as an Anti-obesity Medicine in Individuals Without Diabetes: A Systematic Review and Meta-analysis

**DOI:** 10.17925/EE.2024.20.2.10

**Published:** 2024-06-06

**Authors:** Deep Dutta, A.B.M. Kamrul-Hasan, Lakshmi Nagendra, Saptarshi Bhattacharya

**Affiliations:** 1. Department of Endocrinology, CEDAR Superspeciality Healthcare, Dwarka, New Delhi, India; 2. Department of Endocrinology, Mymensingh Medical College, Mymensingh, Bangladesh; 3. Department of Endocrinology, JSS Medical College, JSS Academy of Higher Education Research, Mysore, India; 4. Department of Endocrinology, Indraprastha Apollo Hospitals, New Delhi, India

**Keywords:** Body mass index, meta-analysis, obesity, safety, tirzepatide, weight loss

## Abstract

**Aims:** To date, no meta-analysis has analyzed the efficacy and safety of tirzepatide as an anti-obesity medication in individuals without diabetes. This meta-analysis was undertaken to address this knowledge gap. **Materials and methods:** Electronic databases were searched for randomized controlled trials (RCTs) involving individuals with obesity without diabetes receiving tirzepatide in the intervention arm and placebo in the control arm. The primary outcome was the percentage change in weight from baseline, and the secondary outcomes included the change in weight from baseline; a weight reduction of ≥5%, ≥10%, ≥15%, ≥20% and ≥25%; glycaemic parameters; lipid parameters and adverse events. **Results:** From 281 initially screened articles, data from 2 RCTs involving 1,852 participants were analyzed. The efficacy and safety of tirzepatide 15 mg (or the highest tolerable dose) versus placebo were analyzed. The percentage change in body weight was higher with tirzepatide than with placebo (mean difference [MD]: -19.44%; 95% confidence interval [CI]: -22.48 to -16.41; p<0.00001). Tirzepatide also had a higher absolute reduction in body weight (MD: -17.55 kg; 95% CI: -32.15 to -2.95; p<0.00001). Higher percentages of people on tirzepatide had a weight reduction of ≥5%, ≥10%, ≥15%, ≥20% and ≥25% compared with placebo. Improvements in glycaemic and cardiometabolic parameters were observed with tirzepatide. Tirzepatide was associated with a higher number of participants with one or more adverse events, which leads to treatment discontinuation, and severe or serious gastrointestinal events. **Conclusion:** This meta-analysis provides exciting data on the impressive weight loss properties of tirzepatide over 72 weeks of clinical use in individuals with obesity without diabetes.

Tirzepatide is a first-i n-class novel dual glucose-dependent insulinotropic peptide (GIP)/glucagon-like peptide-1 (GLP-1) receptor agonist (twincretin), formulated as a synthetic peptide containing 39 amino acids based on the native GIP.^1^ Tirzepatide has a GIP receptor-binding affinity comparable with native GIP and five times lower GLP-1 receptor affinity than native GLP-1.^1^ Several systematic reviews and meta-analyses (SRMs) have been published, highlighting tirzepatide as the most potent anti-diabetes and weight loss medication available for clinical practice in individuals with type 2 diabetes mellitus (T2DM). One of the earliest SRMs published on tirzepatide in individuals with T2DM analyzing the data from six randomized controlled trials (RCTs) with 3,484 patients demonstrated that tirzepatide use was associated with an additional significant mean lowering of glycated haemoglobin (HbA1c) by -0.75%, weight reduction of -8.63 kg, body mass index (BMI) reduction of -1.82 kg/m^2^ and waist circumference (WC) reduction of -4.43 cm compared with potent established anti-diabetes medications, such as dulaglutide, semaglutide, degludec or glargine.^2^ In a recently published updated SRM analyzing the data from 7,062 patients with diabetes, compared with placebo, tirzepatide significantly reduced body weight (mean difference [MD]: -11.34 kg; 95% confidence interval [CI]: -12.79 to -9.88), BMI (MD: -3.11 kg/m^2^; 95% CI: -4.36 to -1.86) and WC (MD: -7.24 cm; 95% CI -10.12 to -4.36).^3^

Several RCTs have been published evaluating tirzepatide as an anti-obesity medication in people without diabetes.^4,5^ Tirzepatide has recently been approved by the US Food and Drug Administration as an anti-obesity medication in people without diabetes.^6^ Our experience with sodium–glucose cotransporter 2 inhibitors tells us that its weight loss properties are primarily observed in people with T2DM and are less pronounced in people without diabetes. The literature review revealed that no SRM had been published that evaluated the weight loss properties of tirzepatide in people with obesity without diabetes. Conducting such an analysis is crucial to ascertain whether tirzepatide demonstrates efficacy as an anti-obesity medicine in individuals with normoglycaemia, similar to what has been documented in individuals with diabetes. Hence, this SRM aimed to evaluate the efficacy and safety of tirzepatide as an anti-obesity medication in people without diabetes.

## Materials and methods

The recommendations of the Cochrane Handbook for Systematic Reviews of Interventions were strictly followed while carrying out this meta-analysis.^7^ The predefined protocol has been registered in PROSPERO with the registration number CRD42023489471. All RCTs published up to November 2023 were considered. This meta-analysis has been reported following the Preferred Reporting Items for Systematic Reviews and Meta-Analyses (PRISMA; *Supplementary Table S1*).^7^ As ethical approval already exists for individual studies, no separate approval was required for this meta-analysis. The Population, Intervention, Comparison, Outcomes and Study (PICOS) criteria were used to screen and select studies.^8^ The studies needed at least two treatment arms/groups, with one of the groups on tirzepatide and the other group receiving placebo in patients with obesity but without diabetes. The primary outcome was the percentage change in weight from baseline. The secondary outcomes included the change in weight from baseline; a weight reduction of ≥5%, ≥10%, ≥15%, ≥20% and ≥25%; HbA1c, fasting plasma glucose (FPG); WC; hypoglycaemia; lipid parameters and adverse events.

A comprehensive search encompassing MEDLINE (via PubMed), Scopus, Google Scholar, Cochrane Central Register of Controlled Trials, ClinicalTrials.gov and International Clinical Trials Registry Platform was conducted from inception to 10 December 2023. The search strategy used a Boolean approach: (tirzepatide) AND (obesity). Furthermore, an additional meticulous manual search of references within prior meta-analyses, the RCTs incorporated in this study and the relevant journals were performed to identify any recently published or unpublished studies.

Data extraction was carried out independently by two authors using data extraction forms, and the details have been elaborated elsewhere.^9^ The details of dealing with the missing data also have been elaborated elsewhere.^9^ Three authors independently assessed the risk of bias using the risk of bias assessment tool in Review Manager (RevMan) Web 2023 Version (The Cochrane Collaboration, Oxford, UK) software.^10^ The details of the different biases looked into have already been elaborated elsewhere.^9^

For continuous variables, outcomes were expressed as mean differences (MD). The International System of Units (SI) was used for the analysis. The results are presented as odds ratios (ORs), with the corresponding 95% CI for dichotomous outcomes. RevMan Web was used to compare the MD of the different primary and secondary outcomes between tirzepatide and placebo groups of the included studies. The data were pooled as random effect models to analyze the primary and secondary outcomes. The outcomes were expressed as 95% CIs. Forest plots were plotted on the left side of the graph, favouring tirzepatide, and on the right side, favouring placebo, using RevMan Web software. Forest plots that included the data from both RCTs were included in the results. p<0.05 was considered statistically significant.

Heterogeneity was initially assessed by studying the forest plot generated for outcomes. Subsequently, it was analyzed using a chi-squared test on N-1 degrees of freedom, with an alpha value of 0.05 used for the statistical significance and the *I*^2^ test.^11^ The details of interpreting *I*^2^ values have already been elaborated elsewhere.^9^

An overall grading of the evidence (certainty of the evidence) related to each meta-analysis outcome was performed using the Grades of Recommendation, Assessment, Development and Evaluation (GRADE) approach.^12^ The details of how GRADE was used to generate the summary of findings (SOF) table and how the grading of evidence was performed as ‘high’, ‘moderate’ or ‘low’ have been elaborated elsewhere.^9^ Publication bias was assessed by plotting funnel plots.^12^ The presence of one or more of the smaller studies outside the inverted funnel plot signifies significant publication bias.^13^

## Results

### Search results

**Figure 1:**
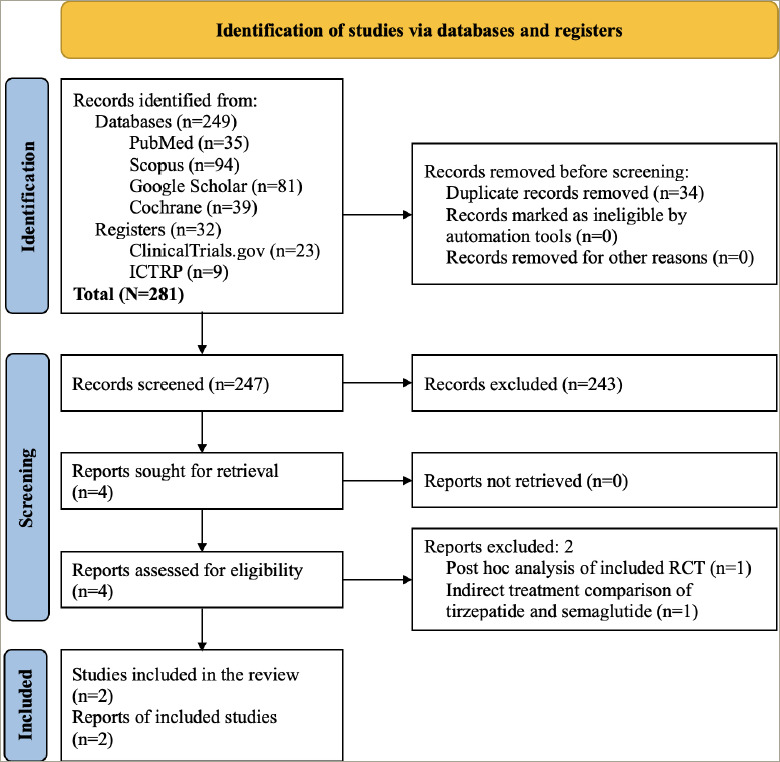
Flowchart of study retrieval and inclusion in the meta-analysis

The study selection process is depicted in *Figure 1*. A total of 281 articles were found after the initial search. After screening the titles and abstracts, followed by full texts, the search was reduced to four studies evaluated in detail for inclusion in this meta-analysis. Finally, two RCTs involving participants with obesity and without diabetes, which fulfilled all the criteria, were analyzed.^4,5^ One of the two excluded studies was the post hoc analysis of an included RCT; the other reported an indirect treatment comparison of tirzepatide and semaglutide.^14,15^

### Study characteristics

Of the two RCTs included in this meta-analysis, one (Efficacy and Safety of Tirzepatide Once Weekly in Participants Without Type 2 Diabetes Who Have Obesity or Are Overweight With Weight-Related Comorbidities: A Randomized, Double-Blind, Placebo-Controlled Trial [SURMOUNT-1]; ClinicalTrials.gov identifier: NCT04184622) has three intervention arms of tirzepatide (5, 10 and 15 mg).^4^ The other study (Efficacy and Safety of Tirzepatide Once Weekly Versus Placebo After an Intensive Lifestyle Program in Participants Without Type 2 Diabetes Who Have Obesity or Are Overweight With Weight-Related Comorbidities: A Randomized, Double Blind, Placebo-Controlled Trial [SURMOUNT-3]; ClinicalTrials.gov identifier: NCT04657016) had one intervention arm of tirzepatide (maximum tolerated dose of 10 or 15 mg).^5^ In this meta-analysis, we used the data from the study participants receiving the highest dose of tirzepatide (15 mg) in the first study. Thus, 1,852 participants (917 in the tirzepatide group and 935 in the placebo group) of the two RCTs were analyzed in this SRM. Both studies had intervention durations of 72 weeks. The details of the included studies are elaborated on in *Table 1*.

### Risk of bias in the included studies

**Figure 2:**
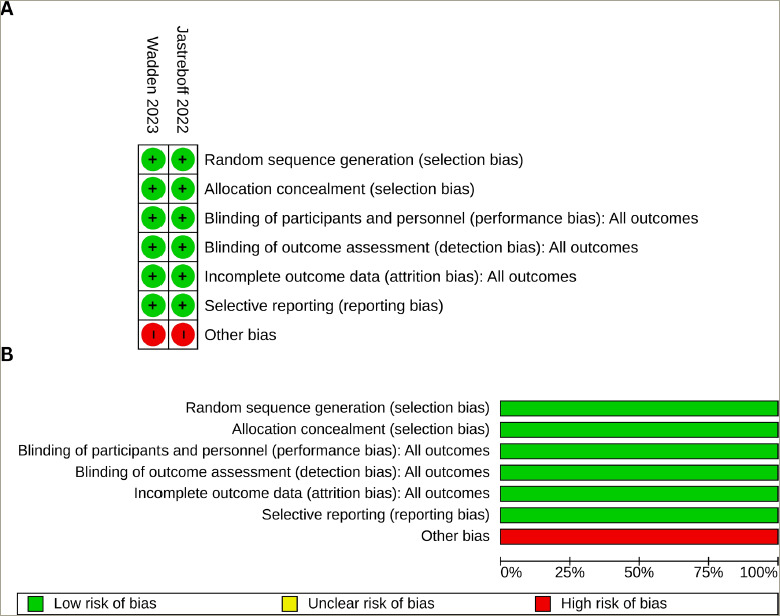
Risk of bias simmary and graph

*Figure 2* depicts the bias risk across the two studies included in the meta-analysis. Both studies (100%) exhibited a low risk of selection bias, performance bias, detection bias, attrition bias and reporting bias; all (100%) had a high risk of other biases. The detailed process of bias risk assessment is available as a supplementary file (*Suppementary Table S1*). Publication bias was assessed through funnel plots given in *Supplementary Figure S1*.

### Effect of tirzepatide on body weight

**Figure 3:**
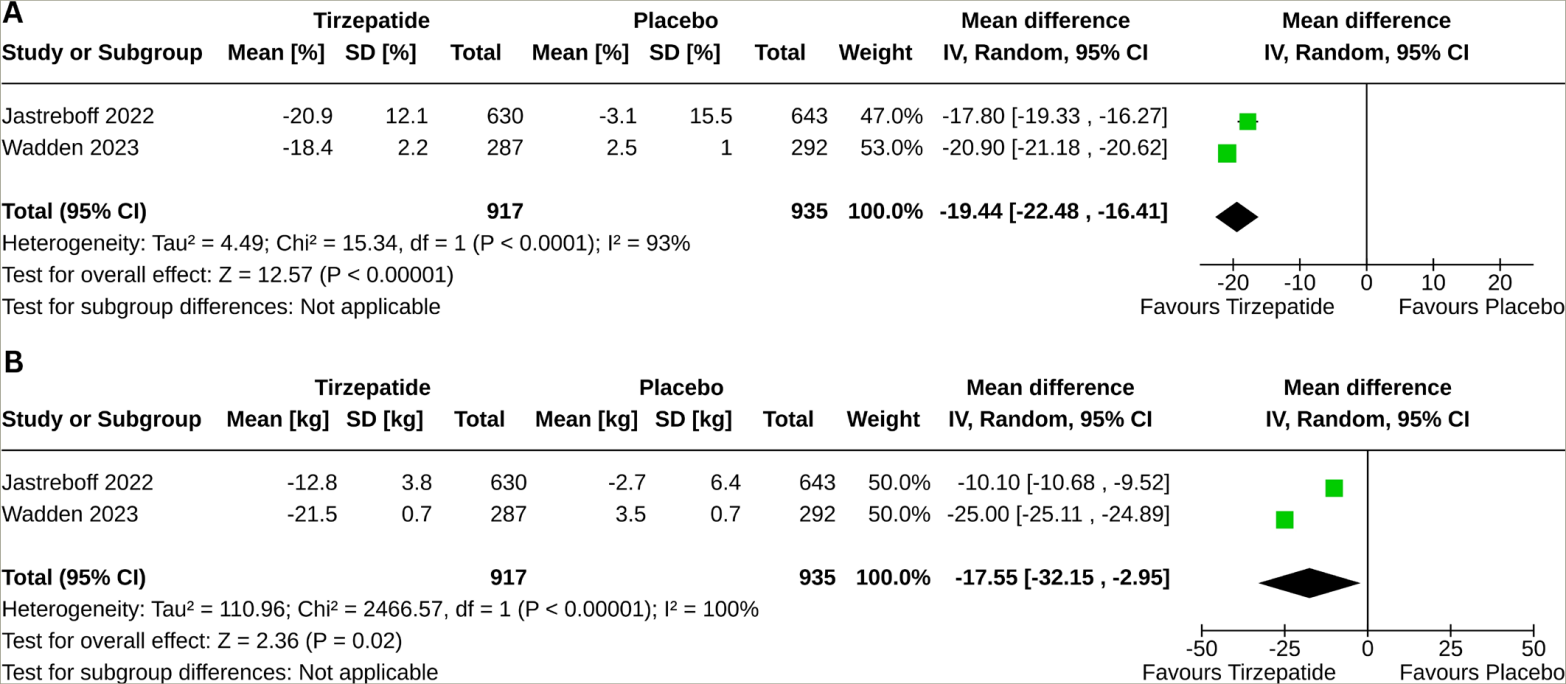
Forest plot highlighting the impact of tirzepatide on body weight

The percentage change in body weight was higher with tirzepatide than with placebo (MD: -19.44%; 95% CI: -22.48 to -16.41; p<0.00001; *I*^2^=93%) (*Figure 3A*); the tirzepatide group also had a higher absolute change in body weight (MD: -17.55 kg; 95% CI: -32.15 to -2.95; p<0.00001; *I*^2^=100%) (*Figure 3B*). Higher percentages of the study participants in the tirzepatide group had a weight reduction of ≥5% (MD: 63.97%; 95% CI: 49.67–78.27; p<0.00001; *I*^2^=96%), ≥10% (MD: 67.38%; 95% CI: 65.29–69.46; p<0.00001; *I*^2^=26%), ≥15% (MD: 61.20%; 95% CI: 60.80–61.61; p<0.00001; *I*^2^=0%), ≥20% (MD: 47.81%; 95% CI: 36.94–58.68; p<0.00001; *I*^2^=96%) and ≥25% (MD: 30.79%; 95% CI: 23.76–37.82; p<0.00001; *I*^2^=91%) than the placebo group (*Table 2*).

*ICTRP = International Clinical Trials Registry Platform; RCT = randomized controlled trial.*

### Effect of tirzepatide on other efficacy outcomes

Compared with placebo, tirzepatide use was associated with higher reductions in WC (MD: -14.80 cm; 95% CI: -14.94 to -14.66; p<0.00001; *I*^2^=0%), systolic blood pressure (BP) (MD: -7.87 mmHg; 95% CI: -10.61 to -5.13; p<0.00001; *I*^2^=95%) and diastolic BP (MD: -4.61 mmHg; 95% CI: -6.47 to -2.75; p<0.00001; *I*^2^=94%). Reductions in HbA1c (MD: -0.47%; 95% CI: -0.52 to -0.41; p<0.00001; *I*^2^=76%) and FPG (MD: -11.20 mg/dL; 95% CI: -11.34 to -11.07; p<0.00001; *I*^2^=0%) were also higher in the tirzepatide group (*Table 2*).

Tirzepatide use was associated with improvements in all the lipid parameters studied, including percentage changes in total cholesterol (MD: -7.47%; 95% CI: -9.28 to -5.66; p<0.00001; *I*^2^=76%), low-density lipoprotein cholesterol (LDL-C) (MD: -10.18%; 95% CI: -14.57 to -5.79; p<0.00001; *I*^2^=90%), high-density lipoprotein cholesterol (HDL-C) (MD: 10.05%; 95% CI: 6.33–13.76; p<0.00001; *I*^2^=92%), non-HDL-C (MD: -13.71%; 95% CI: -17.41 to -10.01; p<0.00001; *I*^2^=89%), very-l ow-density lipoprotein cholesterol (MD: -28.04%; 95% CI: -30.08 to -25.99; p<0.00001; *I*^2^=44%) and triglyceride (TG) (MD: -27.42%; 95% CI: -30.92 to -23.91; p<0.00001; *I*^2^=74%). Percentage changes in free fatty acid (MD: -18.09%; 95% CI: -18.52 to -17.67; p<0.00001; *I*^2^=0%) and fasting insulin (MD: -48.41%; 95% CI: -64.57 to -32.25; p<0.00001; *I*^2^=97%) were also higher in the tirzepatide arm. Finally, the Short Form-36 physical function scores increased more with tirzepatide than with placebo (MD: 3.14; 95% CI: 1.57–4.71; p<0.0001; *I*^2^=95%) (*Table 2*).

**Table 1:** The basic characteristics of the included randomized controlled trials and participants

Authors (year)	Trial name (NCT number)	Major baseline characteristics of the study subjects	Study arms	N	Age (years, mean ± SD)	Female (%)	Baseline body weight (kg, mean ± SD)	Baseline BMI (kg/m^2^, mean ± SD)	RCT duration
Jastreboff et al. (2022)^4^	SURMOUNT-1 (NCT04184622)	-Adults with obesity (BMI >30 kg/m^2^) or overweight (BMI >27 kg/m^2^) with at least one weight-related complication. -Reported one or more unsuccessful dietary effort to lose weight.	Tirzepatide 5 mg once weekly	630	45.6 ± 12.7	67.6	102.9 ±20.71	37.4 ± 6.63	72 weeks
			Tirzepatide 10 mg once weekly	636	44.7 ± 12.4	67.1	105.8 ±23.32	38.2 ±7.01	
			Tirzepatide 15 mg once weekly	630	44.9 ± 12.3	67.5	105.6 ±22.92	38.1 ± 6.69	
			Placebo	643	44.4 ± 12.5	67.8	104.8 ±21.37	28.2 ± 6.89	
Wadden etal. (2023)^5^	SURMOUNT-3 (NCT04657016)	-Adults with obesity (BMI >30 kg/m^2^) or overweight (BMI >27 kg/m^2^) with at least one weight-related complication. -Received intensive lifestyle intervention to achieve >5.0 % body weight reduction for 12 weeks	Tirzepatide 10 or 15 mg once weekly	287	45.4 ± 12.6	63.1	102.5 ±22.1	36.1 ± 6.1	72 weeks
			Placebo once weekly	292	45.7 ± 11.8	62.7	101.3 ±20.7	35.7 ±6.4	

*BMI = body mass index; NCT = National Clinical Trial; RCT = randomized controlled trials; SD = standard deviation.*

*(A) Risk of bias summary: review authors’ judgements about each risk of bias item for each included study; (B) risk of bias graph: review authors’ judgements about each risk of bias item presented as percentages across all the included studies*

### Adverse events

Tirzepatide use was associated with a higher number of participants with one or more adverse events (hazard ratio [HR]: 1.65; 95% CI: 1.19–2.28; p=0.003; *I*^2^=43%), adverse events leading to treatment discontinuation (HR: 3.42; 95% CI: 1.54–7.61; p=0.003; *I*^2^=57%), severe or serious gastrointestinal (GI) events (HR: 3.24; 95% CI: 1.68–6.25; p=0.0005; *I*^2^=0%) and hypoglycaemia (HR: 10.35; 95% CI: 1.32–81.13; p=0.03; *I*^2^=not available [NA]) (*Table 3*).

The risks of serious adverse events, such as pancreatitis, severe or serious acute gallbladder disease, cholelithiasis, severe or serious arrhythmias, cardiac conduction disorders, major adverse cardiovascular events (MACE), renal events, major depressive disorder or suicidal ideation, malignancies and death, were similar in the two groups (*Table 3*).

Percentage reductions in alanine transaminase (ALT) (MD: -18.94%; 95% CI: -21.78 to -16.10; p<0.00001; *I*^2^=99%), aspartate transferase (MD: -8.19%; 95% CI: -9.36 to -7.01; p<0.00001; *I*^2^=97%) and urinary albumin:creatinine ratio (ACR) (MD: -4.83%; 95% CI: -7.37 to -2.28; p=0.0002; *I*^2^=97%) were higher in the tirzepatide group than in the placebo group. Percentage elevations in pancreatic amylase (MD: 19.55%; 95% CI: 14.35–24.74; p<0.00001; *I*^2^=100%) and pancreatic lipase (MD: 34.20%; 95% CI: 24.99–43.41; p<0.00001; *I*^2^=100%) were higher in the tirzepatide group. At the same time, the percentage change in serum calcitonin was similar in the two groups (MD: 4.45%; 95% CI: -11.13 to 20.04; p=0.58; *I*^2^=100%) (*Table 2*). A significant increase in the pulse rate was noted with the use of tirzepatide compared with controls (MD: 2.15%; 95% CI: 1.47–2.84; p<0.00001; *I*^2^=99%) (*Table 3*).

High publication bias was noted for percentage reductions in body weight, absolute reduction in body weight, ≥5% weight loss, ≥10% weight loss and ≥25% weight loss, as one of the studies was outside the funnel plot (*Supplementary Figure S1*). Low publication bias was noted for participants with ≥10% body weight loss, ≥15% body weight loss, one or more adverse events, adverse events leading to treatment discontinuation, severe or serious GI events, pancreatitis, severe or acute gall bladder disease, cholelithiasis, severe or serious arrhythmias and cardiac conduction disorders and severe hypoglycaemia (*Supplementary Figure S1*).

### Grading of the results

The grades of the certainty of evidence of some of the key outcomes of this study have been elaborated in the SOF table (*Table 4*). The currently available percentage weight loss and absolute weight loss data with tirzepatide in obesity without diabetes are of low grade due to significant data heterogeneity and publication bias. However, the side-effect profile and safety data of tirzepatide in obesity without diabetes are of high grade (*Table 4*).

*Forest plot highlighting the impact of tirzepatide on (A) the percentage change in body weight and (B) the absolute change in body weight from baseline compared with placebo CI = confidence interval; SD = standard deviation.*

## Discussion

Tirzepatide has emerged as a transformative breakthrough in the management of obesity. A remarkable achievement associated with tirzepatide is the attainment of a weight loss exceeding 15%, a milestone strongly linked to the remission of diabetes and the discontinuation of anti-diabetes medications, particularly in individuals with recent-onset obesity lasting less than 5 years.^16^ Historically, achieving such a significant weight loss was primarily observed with metabolic surgeries or very-l ow-calorie diets, both of which pose challenges in terms of sustainability over prolonged periods. However, tirzepatide has demonstrated the potential for greater than 15% weight loss in individuals with obesity.^17^ Several factors have been identified as predictors of achieving this substantial weight loss with tirzepatide, including higher tirzepatide doses, female sex, individuals of White or Asian race, younger age, background therapy with metformin, better glycaemic status and lower non-HDL-C. This signifies a noteworthy advancement in the therapeutic options for managing obesity, offering a more accessible and sustainable approach to significant weight loss.^17^

**Table 2:** Summary of the secondary outcome findings

	Sample size			
Outcome variables		Tirzepatide arm	Placebo arm	*I*^2^ (%)	Pooled effect size, MD (95% CI)	p-value
Participants achieving ≥5% body weight reduction	917	935	96	63.97 (49.67–78.27)	<0.00001
Participants achieving ≥10% body weight reduction	917	935	26	67.38 (65.29–69.46)	<0.00001
Participants achieving ≥15% body weight reduction	917	935	0	61.20 (60.80–61.61)	<0.00001
Participants achieving ≥20% body weight reduction	917	935	96	47.81 (36.94–58.68)	<0.00001
Participants achieving ≥25% body weight reduction	917	935	91	30.79 (23.76–37.82)	<0.00001
Waist circumference (cm)	917	935	0	-14.80 (-14.94 to -14.66)	<0.00001
Systolic BP (mmHg)	917	935	95	-7.87 (-10.61 to -5.13)	<0.00001
Diastolic BP (mmHg)	917	935	94	-4.61 (-6.47 to -2.75)	<0.00001
Percentage change in pulse (beats/min)	917	935	99	2.15 (1.47–2.84)	<0.00001
HbA1c (%)	917	935	76	-0.47 (-0.52 to -0.41)	<0.00001
FPG (mg/dL)	917	935	0	-11.20 (-11.34 to -11.07)	<0.00001
Percentage change in TC (mg/dL)	917	935	76	-7.47 (-9.28 to -5.66)	<0.00001
Percentage change in LDL-C (mg/dL)	917	935	90	-10.18 (-14.57 to -5.79)	<0.00001
Percentage change in HDL-C (mg/dL)	917	935	92	10.05 (6.33–13.76)	<0.00001
Percentage change in non-HDL-C (mg/dL)	917	935	89	-13.71 (-17.41 to -10.01)	<0.00001
Percentage change in VLDL-C (mg/dL)	917	935	44	-28.04 (-30.08 to -25.99)	<0.00001
Percentage change in TG (mg/dL)	917	935	74	-27.42 (-30.92 to -23.91)	<0.00001
Percentage change in FFA (mmol/L)	917	935	0	-18.09 (-18.52 to -17.67)	<0.00001
Percentage change in fasting insulin (mIU/L)	917	935	97	-48.41 (-64.57 to -32.25)	<0.00001
SF-36 physical function score	917	935	95	3.14 (1.57–4.71)	<0.0001

*BP = blood pressure; CI = confidence interval; df = degree of freedom; FFA = free fatty acid; FPG = fasting plasma glucose; HbA1c = glycated haemoglobin; HDL-C = high-density lipoprotein cholesterol; IV = inverse variance; LDL-C = low-density lipoprotein cholesterol; MD = mean difference; SF-36 = Short Form-36; TC = total cholesterol; TG = triglyceride; VLDL-C = very-low-density lipoprotein cholesterol.*

**Table 3:** The results of safety in the meta-analysis

	Number of participants with outcome/participants analysed			
Safety variables		Tirzepatide arm	Placebo arm	*I*^2^ (%)	Pooled effect size, HR (95% CI)	p Value
Participants with one or more adverse events	747/917	687/935	43	1.65 (1.19–2.28)	0.003
Adverse events leading to treatment discontinuation	69/917	23/935	57	3.42 (1.54–7.61)	0.003
Serious adverse events	49/917	58/935	33	0.89 (0.53–1.47)	0.64
Severe or serious gastrointestinal events	37/917	12/935	0	3.24 (1.68–6.25)	0.0005
Pancreatitis	2/917	2/935	0	1.02 (0.14–7.25)	0.98
Severe or serious acute gallbladder disease	8/917	5/935	0	1.48 (0.49–4.50)	0.49
Cholelithiasis	8/917	9/935	0	0.91 (0.34–2.39)	0.84
Severe or serious arrhythmias and cardiac conduction disorders	2/917	2/935	0	1.07 (0.16–7.32)	0.95
MACE	1/917	6/935	27	0.32 (0.03–3.34)	0.34
Renal events	3/917	1/935	0	2.36 (0.35–16.17)	0.38
Hypoglycaemia	10/917	1/935	NA	10.35 (1.32–81.13)	0.03
Major depressive disorder or suicidal ideation	3/917	0/935	0	4.01 (0.44–36.41)	0.22
Malignancies	10/917	10/935	0	1.01 (0.41–2.50)	0.97
Death	2/917	5/935	0	0.43 (0.08–2.42)	0.34
				MD (95% CI)	
Percentage change in ALT	917	935	99	-18.94 (-21.78 to -16.10)	<0.00001
Percentage change in AST	917	935	97	-8.19 (-9.36 to -7.01)	<0.00001
Percentage change in pancreatic amylase	917	935	100	19.55 (14.35–24.74)	<0.00001
Percentage change in pancreatic lipase	917	935	100	34.20 (24.99–43.41)	<0.00001
Percentage change in calcitonin	917	935	100	4.45 (-11.13 to 20.04)	0.58
Percentage change in urinary ACR	917	935	97	-4.83 (-7.37 to -2.28)	0.0002

*ACR = albumin:creatinine ratio; ALT = alanine transaminase; AST = aspartate transferase; CI = confidence interval; HR = hazard ratio; MACE = major adverse cardiovascular events; MD = mean difference.*

Our SRM underscores the sustained effectiveness of tirzepatide as a potent weight loss medication in individuals with obesity but without diabetes. Notably, tirzepatide at the maximum tolerated dose exhibited an impressive average weight reduction of 19.44%, equivalent to an absolute weight loss of 17.55 kg, over 18 months of clinical use. Analyzing the data reveals compelling results: individuals with obesity on tirzepatide achieved noteworthy weight-l oss percentages, with averages of 63.97%, 67.38%, 61.2%, 47.81% and 30.79 % for ≥5%, ≥10%, ≥15%, ≥20% and ≥25%, respectively. These results parallel those reported for bariatric surgeries, such as sleeve gastrectomy.^18^ The magnitudes of weight loss with tirzepatide 15 mg in the previous meta-analyses were lower (Tan et al., -11.77 kg, 95% CI: -17.12 to -6.43; Lin et al., -10.91 kg, 95% CI: -14.81 to -7.01; Cai et al., -10.28 kg, 95% CI: -14.34 to -6.23) than the current meta-analysis.^19–21^ Previous meta-analyses included RCTs conducted on participants who were overweight or obese, irrespective of whether they had T2DM or not. Weight loss medications cause a greater extent of weight loss in participants with obesity without T2DM than in participants with obesity and T2DM.^22^ Lin et al. and Cai et al. included the SURMOUNT-1 trial, which was also included in this meta-analysis.^20,21^ None of these meta-analyses included the SURMOUNT-3 trial that was included in the present meta-analysis.

Beyond the significant weight loss, tirzepatide demonstrated pleiotropic benefits. These include a mean reduction in WC by 14.8 cm, a decrease in systolic BP by 7.87 mmHg, a lowering of diastolic BP by 4.61 mmHg, an average reduction in HbA1c by 0.47%, a decrease in FPG by an average of 11.2 mg/dL, a substantial 27.42% reduction in TG, a 10.18% reduction in LDL-C, an 18.94% reduction in ALT (a measure of steatotic liver disease) and a 4.83% reduction in urine ACR (indicative of endothelial dysfunction and renal involvement). Furthermore, the mean decrease in total body fat mass was higher with tirzepatide than with the placebo, and the ratio of total fat mass to total lean mass decreased more with tirzepatide than with the placebo.^4^ The multifaceted mechanisms contributing to this weight loss with tirzepatide include delayed gastric emptying, reduced food intake due to early satiety and GI and central effects. This comprehensive set of outcomes positions tirzepatide as a promising and versatile intervention for obesity management with far-reaching benefits beyond weight reduction alone.

**Table 4:** Summary of findings of the key outcomes of this systematic review and meta-analysis

	Anticipated absolute effects‡ (95% CI)			
Outcomes		Risk with placebo	Risk with tirzepatide	Relative effect (95% CI)	Number of participants (studies)	Certainty of the evidence (GRADE)
Percentage change in body weight	The mean percentage change in body weight was -1.35%	MD 20.8 lower (21.07 lower to 20.52 lower)	-	1,852 (2 RCTs)	⨁⨁◯◯ Low*†
Absolute change in body weight	The mean absolute change in body weight was -0.76 kg	MD 24.44 lower (24.55 lower to 24.33 lower)	-	1,852 (2 RCTs)	⨁⨁◯◯ Low*†
Participants achieving ≥5% body weight reduction	The mean participants achieving ≥5% body weight reduction was 36.03%	MD 70.91 higher (70.49 higher to 71.34 higher)	-	1,852 (2 RCTs)	⨁⨁◯◯ Low*†
Participants achieving ≥10% body weight reduction	The mean participants achieving ≥10% body weight reduction was 29.12%	MD 67.38 higher (65.29 higher to 69.46 higher)	-	1,852 (2 RCTs)	⨁⨁⨁◯ Moderate†
Participants achieving ≥15% body weight reduction	The mean participants achieving ≥15% body weight reduction was 26.64%	MD 61.2 higher (60.8 higher to 61.61 higher)	-	1,852 (2 RCTs)	⨁⨁⨁⨁ High
Participants achieving ≥20% body weight reduction	The mean participants achieving ≥20% body weight reduction was 17.63%	MD 47.81 higher (36.94 higher to 58.68 higher)	-	1,852 (2 RCTs)	⨁⨁⨁◯ Moderate*
Participants achieving ≥25% body weight reduction	The mean participants achieving ≥25% body weight reduction was 12.39%	MD 30.79 higher (23.76 higher to 37.82 higher)	-	1,852 (2 RCTs)	⨁⨁◯◯ Low*†
Participants with one or more adverse events	735 per 1,000	820 per 1,000 (767–863)	OR 1.65 (1.19–2.28)	1,852 (2 RCTs)	⨁⨁⨁⨁ High
Adverse events leading to treatment discontinuation	25 per 1,000	79 per 1,000 (46–113)	OR 3.42 (1.54–7.61)	1,852 (2 RCTs)	⨁⨁⨁⨁ High
Severe or serious gastrointestinal events	13 per 1,000	40 per 1,000 (21–75)	OR 3.24 (1.68–6.25)	1,852 (2 RCTs)	⨁⨁⨁⨁ High
Pancreatitis	2 per 1,000	2 per 1,000 (0–15)	OR 1.02 (0.14–7.25)	1,852 (2 RCTs)	⨁⨁⨁⨁ High
Severe or serious acute gallbladder disease	5 per 1,000	8 per 1,000 (3–24)	OR 1.48 (0.49–4.50)	1,852 (2 RCTs)	⨁⨁⨁⨁ High
Cholelithiasis	10 per 1,000	9 per 1,000 (3–23)	OR 0.91 (0.34–2.39)	1,852 (2 RCTs)	⨁⨁⨁⨁ High
Severe or serious arrhythmias and cardiac conduction disorders	2 per 1,000	2 per 1,000 (0–15)	OR 1.07 (0.16–7.32)	1,852 (2 RCTs)	⨁⨁⨁⨁ High
Hypoglycaemia	1 per 1,000	11 per 1,000 (1–80)	OR 10.35 (1.32–	81.13)	1,852 (2 RCTs)	⨁⨁⨁⨁ High

*Significant heterogeneity in data present as I^2^ >90%.

*†Presence of one study outside the funnel plot (see the supplementary figure).*

*‡The risk in the intervention group (and its 95% CI) is based on the assumed risk in the comparison group and the relative effect of the intervention (and its 95% CI). CI = confidence interval; MD = mean difference; OR = odds ratio; RCT = randomized controlled trial.*

To some extent, the mechanism of action of tirzepatide explains its predominant GI side-effect profile. In our analysis, we noted that people on tirzepatide were 1.65-, 3.42-and 3.24-times more likely to have one or more adverse events, adverse events leading to treatment discontinuation and severe GI events, respectively, compared with placebo. Blood glucose monitoring may be warranted even in individuals with obesity without diabetes, as an increased occurrence of hypoglycaemia has been noted in this SRM.

The limitations on the use of the full dose of tirzepatide primarily stem from its notable GI side effects. In a recently published SRM analyzing the data from 4,586 patients with T2DM, the occurrence of nausea (20.43% versus 10.47%; risk ratio [RR]: 2.90; 95% CI: 1.89–4.44), vomiting (9.05% versus 4.86%; RR: 2.69; 95% CI: 1.67–4.36), constipation (2.54% versus 0.85%; RR: 3.08; 95% CI: 1.83–5.20), diarrhoea (16.24% versus 8.63%; RR: 2.07; 95% CI: 1.60–2.68), decreased appetite (9.64% versus 2.88%; RR: 5.04; 95% CI: 3.01–8.45) and dyspepsia (7.13% versus 3.31%; RR: 2.52; 95% CI: 1.58–4.01) was significantly higher with tirzepatide compared with controls.^23^

No increased occurrence of serious adverse events, such as pancreatitis, serious acute gallbladder disease, cholelithiasis, arrhythmias (despite an increase in heart rate), major depressive disorder or suicidal ideation, malignancies or death, was noted with tirzepatide.^24^ An asymptomatic elevation of pancreatic amylase by 19.55% and pancreatic lipase by 34.20% was recorded with tirzepatide, which did not translate into an increased occurrence of pancreatitis. In another SRM analyzing the data from nine studies with 9,871 participants, tirzepatide use was not associated with increased pancreatitis (RR: 1.46; 95% CI: 0.59–3.61).^24^ However, the authors noted that the gallbladder or biliary disease composite was significantly higher with tirzepatide use than with placebo or basal insulin (RR: 1.97; 95% CI: 1.14–3.42).^24^ As gall bladder and biliary disease tend to be more common in people with obesity, caution needs to be exercised about the use of tirzepatide in these patients. It would be a reasonably good clinical practice not to use tirzepatide in individuals with obesity who have had a history of pancreatitis (of any cause) and in people with active gall bladder disease, similar to the recent history of cholecystitis or active cholelithiasis. However, once these patients have undergone cholecystectomy, the use of tirzepatide should not pose any issues.

A significant increase in heart rate was noted in this SRM, as has previously been reported in people living with T2DM.^24^ Heart rate increase with tirzepatide was reportedly dose-dependent and higher than other GLP-1 receptor agonists, and the reason remains to be determined.^25^ Hence, it would be a good clinical practice to avoid the use of tirzepatide in people with tachyarrhythmias, which again tends to be more common in people with obesity.

Weight loss has consistently been associated with improved fertility.^26^ The use of tirzepatide and GLP1 receptor agonists has been associated with decreased GI absorption of oral contraceptive pills (OCPs), thus reducing their efficacy as contraceptives.^27^ Hence, women with obesity on tirzepatide should be encouraged to switch over to alternative methods of contraception, such as barrier methods, instead of OCPs. Real-world studies have documented reduced craving reduction, decreased desire to drink and an overall reduction in alcohol consumption in people with obesity and alcohol use disorder on tirzepatide or semaglutide.^28^ Our analysis also noted a nearly 4.45% increase in circulating serum calcitonin levels. No human study to date has reported an increased occurrence of medullary thyroid carcinoma with tirzepatide. However, it remains a good clinical practice to completely avoid the use of tirzepatide-and incretin-based therapies in people with a history of medullary thyroid carcinoma and multiple endocrine neoplasia syndromes.

### Strengths and limitations

The key strength of this review is that it is the first SRM to analyze the weight loss potential of tirzepatide in individuals with obesity but without diabetes. An SRM is as good as the quality of the RCTs analyzed. Both the RCTs analyzed in this SRM are of high quality, contributing to the robustness of the findings. Additionally, including many patients in both studies further strengthens the validity of the conclusions. A notable limitation of this SRM is the absence of an analysis of the data from diverse ethnic groups across different regions of the globe. This limitation arises due to the scarcity of the available data for analysis.

## Conclusions

To conclude, this SRM provides us with exciting data on the impressive weight loss properties of tirzepatide after 72 weeks of clinical use in individuals with obesity and without diabetes. Further long-term studies are needed to establish the long-term durability of weight loss with tirzepatide use over several years.
